# Adipose microenvironment promotes triple negative breast cancer cell invasiveness and dissemination by producing CCL5

**DOI:** 10.18632/oncotarget.8336

**Published:** 2016-03-24

**Authors:** Vittoria D’Esposito, Domenico Liguoro, Maria Rosaria Ambrosio, Francesca Collina, Monica Cantile, Rosa Spinelli, Gregory Alexander Raciti, Claudia Miele, Rossella Valentino, Pietro Campiglia, Michelino De Laurentiis, Maurizio Di Bonito, Gerardo Botti, Renato Franco, Francesco Beguinot, Pietro Formisano

**Affiliations:** ^1^ Department of Translational Medicine, Federico II University of Naples and URT “Genomic of Diabetes” of Institute of Experimental Endocrinology and Oncology, National Council of Research (CNR), Naples, Italy; ^2^ Pathology Unit, National Institute of Tumors, Fondazione “G. Pascale”, Naples, Italy; ^3^ Department of Pharmacy, University of Salerno, Salerno, Italy; ^4^ Department of Breast Surgery and Cancer Prevention; National Institute of Tumors, Fondazione “G. Pascale”, Naples, Italy

**Keywords:** triple negative breast cancer, adipose tissue, cytokines, diabetes, obesity

## Abstract

Growing evidence indicates that adiposity is associated with raised cancer incidence, morbidity and mortality. In a subset of tumors, cancer cell growth and/or metastasis predominantly occur in adipocyte-rich microenvironment. Indeed, adipocytes represent the most abundant cell types surrounding breast cancer cells. We have studied the mechanisms by which peritumoral human adipose tissue contributes to Triple Negative Breast Cancer (TNBC) cell invasiveness and dissemination.

Co-culture with human adipocytes enhanced MDA-MB231 cancer cell invasiveness. Adipocytes cultured in high glucose were 2-fold more active in promoting cell invasion and motility compared to those cultured in low glucose. This effect is induced, at least in part, by the CC-chemokine ligand 5 (CCL5). Indeed, CCL5 inhibition by specific peptides and antibodies reduced adipocyte-induced breast cancer cell migration and invasion. CCL5 immuno-detection in peritumoral adipose tissue of women with TNBC correlated with lymph node (*p*-value = 0.04) and distant metastases (*p*-value = 0.001). A positive trend was also observed between CCL5 expression and glycaemia. Finally, Kaplan-Meier curves showed a negative correlation between CCL5 staining in the peritumoral adipose tissue and overall survival of patients (*p*-value = 0.039).

Thus, inhibition of CCL5 in adipose microenvironment may represent a novel approach for the therapy of highly malignant TNBC.

## INTRODUCTION

Adipose tissue represents a major component of the tumor microenvironment, particularly for breast cancer [1, 2]. Beside traditionally considered as an insulating and mechanically supportive site of energy storage, adipose tissue has endocrine functions, capable of regulating systemic energy and metabolic homeostasis through a complex network of signals [2, 3]. The concept that adipose tissue, and more specifically adipocytes, are involved in tumor initiation, growth, and metastasis, is now called “adiponcosis” [4].

Adipocytes surround breast cancer cells and may contribute to the stromal–ductal epithelial cell–cell interactions within the mammary microenvironment [5]. In this regard, obesity has been shown to increase rates of breast cancer in postmenopausal women by 30–50% [1]. Studies of mortality and survival illustrate that adiposity is associated with both poorer survival and increased likelihood of recurrence among breast cancer cases, regardless of menopausal status and after adjustment for stage and treatment. Death rate in very obese women (BMI ≥ 40.0) with breast cancer is three times higher than in very lean (BMI < 20.5) women [6].

Moreover, epidemiological studies have also revealed that women with diabetes have a statistically significant 20% increased risk of breast cancer [7, 8]. Additionally, patients with breast cancer and preexisting diabetes have increased risk for distant metastasis and for all-cause mortality compared with their non-diabetic counterparts [9].

Metabolic derangements, such as obesity and type 2 diabetes, drive to adipocyte alterations with imbalanced production of adipokines, proinflammatory cytokines, chemokines, growth factors, hormones, proangiogenic factors and extracellular matrix constituents [10]. Virtually, all of the adipocyte factors may be envisioned as contributing factors for cancer onset and/or progression. *In vitro* and *in vivo* studies demonstrated that adipocytes could promote breast tumor growth [5, 11]. Moreover, glucose and fatty acids modify adipocyte-releasing properties and enhance their ability to induce breast cancer cell proliferation [11, 12]. Nevertheless, how metabolic alterations at the level of the adipose tissue may affect tumor progression is still unclear.

Here, we show that adipocytes may integrate inputs from metabolic environment and promote motility and invasiveness of breast cancer cells. This effect is induced, at least in part, by the CC-chemokine ligand 5 (CCL5), also known as RANTES (Regulated upon Activation, Normal T-cell Expressed and Secreted), whose abundance in peritumoral adipose tissue correlates with metastasis and with poorer overall survival in women with Triple Negative Breast Cancer (TNBC).

## RESULTS

### Adipocytes promote TNBC cell motility

**Figure 1:**
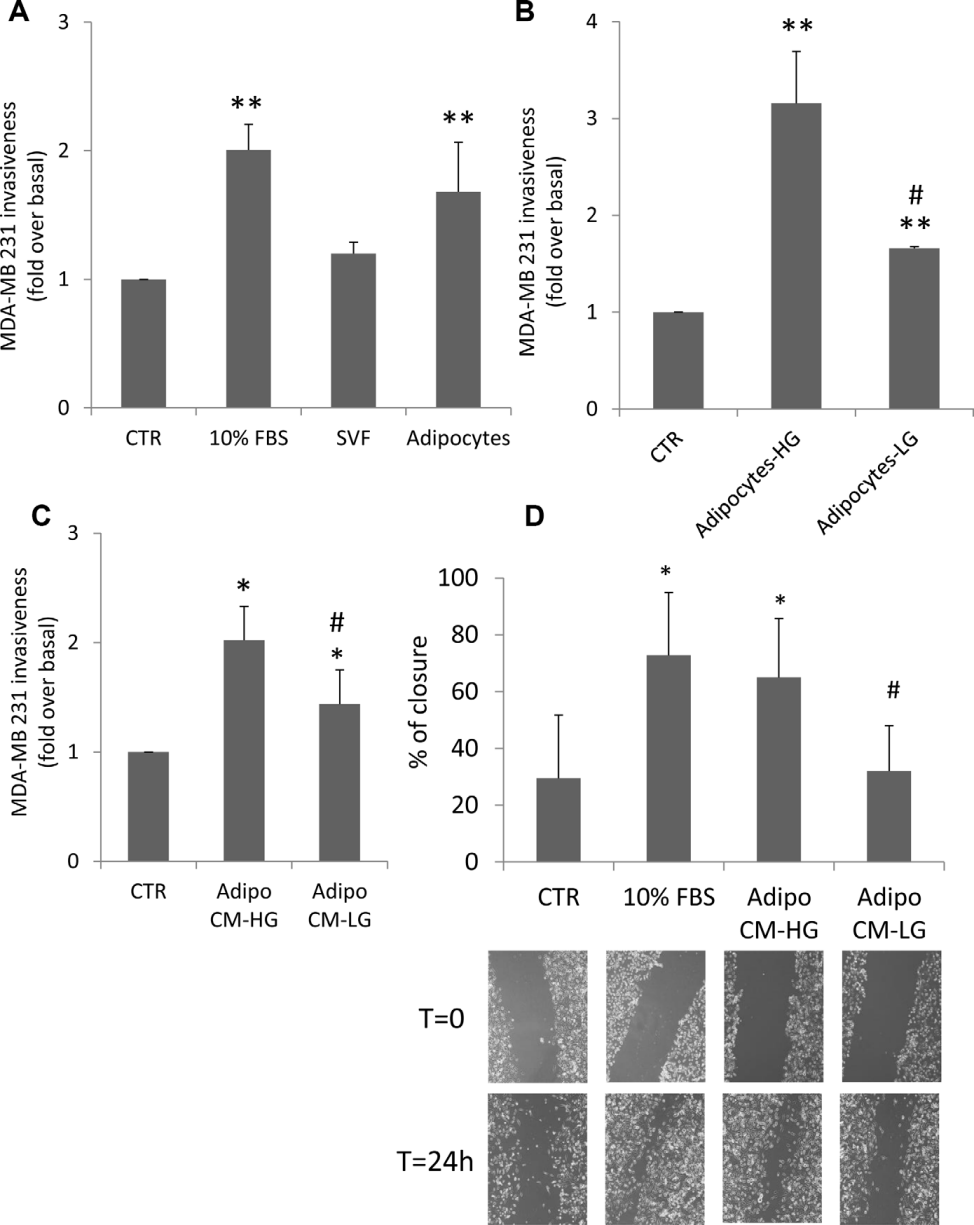
Effect of adipocytes and glucose-treated adipocytes on breast cancer cell motility (**A**) MDA-MB231 cells were seeded in the upper chamber of a matrigel-coated transwell culture system with or without human SVF/adipocytes in the lower chamber in serum free-medium. Cells that migrated across the matrigel-coated filter were determined by crystal violet staining as described in Materials and Methods. The results have been reported as percentage of stained cells compared to control cells (MDA-MB231 cells without SVF/adipocytes in the lower chamber, incubated in serum-free medium). *denote statistically significant values (**p < 0.01). (**B**) Human adipocytes were shifted with high glucose medium (25 mM glucose-HG) or with low glucose medium (5.5 mM glucose-LG) for 24 h. Then, they were incubated with MDA-MB231 cells seeded in the upper chamber of a matrigel-coated transwell culture system. Cancer cells that migrated across the matrigel-coated filter were determined by crystal violet staining. The results have been reported as percentage of stained cells compared to control cells (MDA-MB231 cells incubated in serum-free HG or LG medium). *denote statistically significant values over control (**p < 0.01). ^#^denote statistically significant values of HG over LG (^#^*p* < 0.05). (**C**) Human adipocytes were pre-incubated with HG medium or with LG medium for 24 h. Then, they were further incubated with regular glucose (15 mM) serum-free medium for 8 h. Media were collected (CM) and applied in the lower chamber of a matrigel-coated transwell culture system with MDA-MB231 cells seeded in the upper chamber. Cancer cells that migrated across the matrigel-coated filter were determined by crystal violet staining. The results have been reported as percentage of stained cells compared to control cells (MDA-MB231 cells incubated in regular glucose serum-free medium). *denote statistically significant values over control (*p < 0.05). ^#^denote statistically significant values of CM-HG over CM-LG (^#^*p* < 0.05). (**D**) Confluent monolayers of MDA-MB231 were wounded by manually scratching as described in Materials and Methods and incubated with CM collected from human adipocytes pre-incubated with either LG or HG medium for 24 h and further incubated with regular glucose serum-free medium for 8 h. Images of wound gap were taken at 0 and 24 h by a digital camera coupled to the microscope and percentage of wound distance was calculated with the camera software. The results have been reported as percentage of gap closure at 24 h compared with time 0. A complete gap closure was considered as 100%. *denote statistically significant values over MDA-MB231 cells incubated in regular glucose serum-free medium (*p < 0.05). ^#^denote statistically significant values of CM-HG over CM-LG (^#^*p* < 0.05). The pictures are representative of wound gaps at 0 point and upon 24 h of scratch assay. For all the panels in the figure, data in the graphs represent the mean ± SD of at least three independent triplicate experiments.

In order to investigate adipocyte effect on cancer cell invasiveness, MDA-MB231 triple negative breast cancer cells were seeded in the upper chamber of a matrigel-coated transwell, while differentiated human adipocytes were seeded in the lower chamber. Co-culture with adipocytes, in the absence of serum, increased MDA-MB231 invasive capacity through the matrigel filter by 1.7-fold compared to the same cells cultured in the absence of adipocytes (Figure 1A). At variance, co-culture with human Stromal Vascular Fraction (SVF) cells enhanced by only 1.2-fold MDA-MB231 invasion. The effect of adipocyte factors was similar to the positive control (i.e. cells incubated with 10% FBS medium).

Next, we tested whether glucose may change the promoting action of human adipocytes on MDA-MB231 invasiveness. To this end, human differentiated adipocytes, regularly cultured in 15 mM glucose, were shifted for 24 h in either 25 mM glucose (HG), a concentration resembling hyperglycemia in humans, or in 5.5 mM glucose (LG), a concentration representative of normal fasting glucose levels in humans. Then, adipocytes were co-cultured with MDA-MB231 in serum-free HG or LG medium for additional 24 h. As shown in Figure 1B adipocytes significantly increased cancer cell invasiveness and this effect is potentiated in HG (3-fold increase compared to LG). Similar results were obtained also with conditioned media (CM) system. In detail, adipocytes were cultured for 24 h either in HG and in LG. Media were changed and cells were allowed to secrete factors into freshly added serum free medium (15 mM glucose). After 8 h, CM were collected and applied into the lower chamber of a transwell system in presence of MDA-MB231 cells seeded in the upper chamber on a matrigel-coated filter. As shown, pre-incubation of adipocytes with HG medium enhanced by about 2-fold their ability to induce MDA-MB231 cell invasiveness, compared to control cells (without CM) (Figure 1C). At variance, pre-incubation with LG medium significantly lowered their ability to promote invasiveness of breast cancer cells (Figure 1C).

In order to test cell motility, confluent monolayers of MDA-MB231 were wounded longitudinally and incubated with conditioned media derived from adipocytes incubated with HG (HG-CM) or LG (LG-CM) in presence of mitomycin C, an irreversible inhibitor of mitosis. Images were taken at 0 and 24 h after wounding. HG-CM increased motility of breast cancer cells by about 2-fold (Figure 1D). The wound closure was similar to that achieved with 10% FBS medium and significantly higher compared to that observed with LG-CM. Similar results were also obtained with ER-α positive MCF-7 breast cancer cells (Supplementary Figure 1).

### Adipocyte-released CCL5 promotes motility and invasion of breast cancer cells

**Figure 2:**
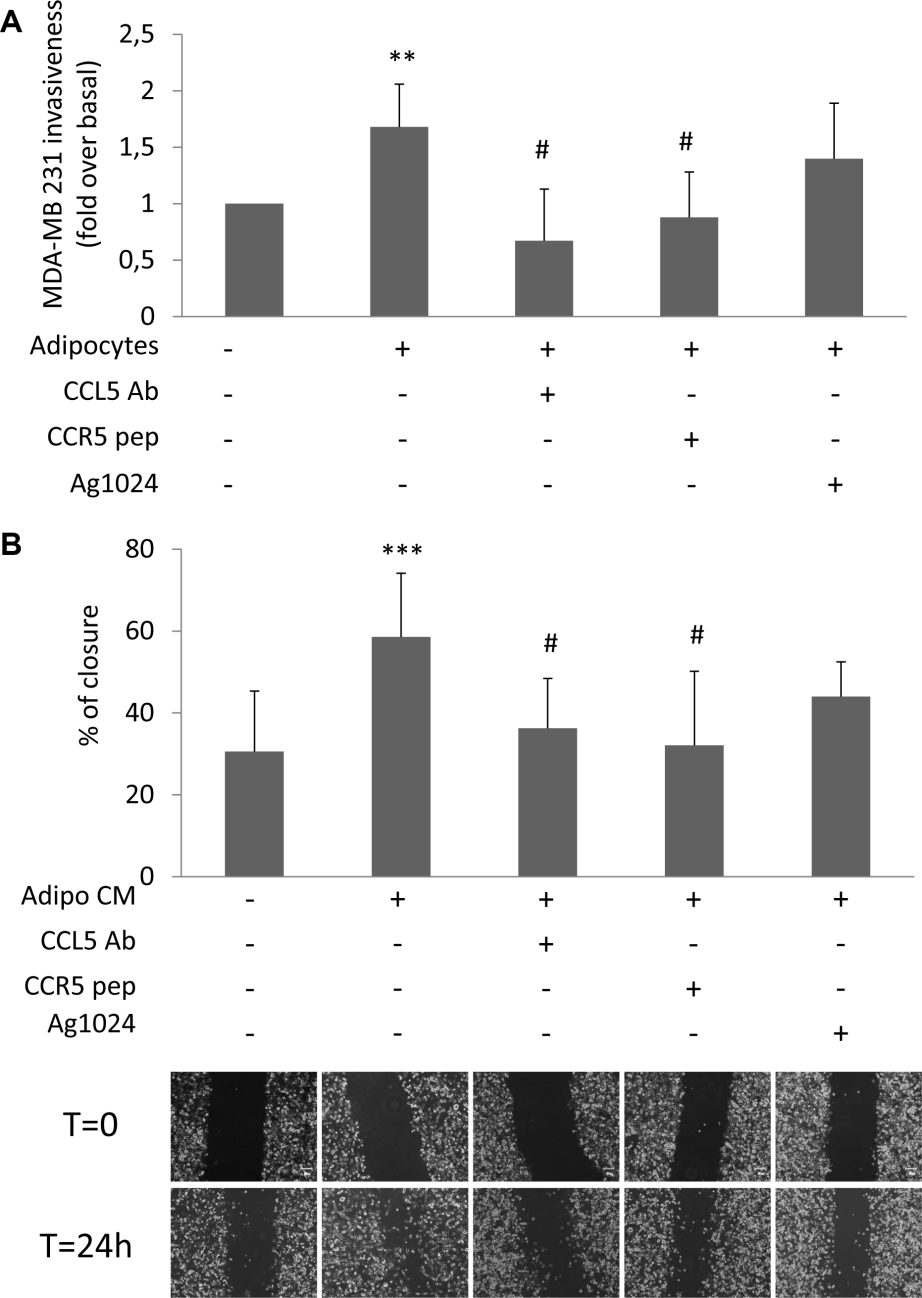
Effect of CCL5 and IGF-1 pathway inhibition on adipocyte-induced breast cancer cell motility (**A**) MDA-MB231 cells were seeded in the upper chamber of a matrigel-coated transwell culture system with or without human adipocytes in the lower chamber in serum free-medium. Co-cultured cells were incubated with 6 μg/ml CCL5 Antibody (CCL5 Ab), 5 μg/ml CCR5 peptide (CCR5 pep) or 10 μM AG1024. Cancer cells that migrated across the matrigel-coated filter were determined by crystal violet staining. The results have been reported as percentage of stained cells compared to control cells (MDA-MB231 cells without adipocytes in the lower chamber, incubated in serum-free medium). *denote statistically significant values over control (**p < 0.01). ^#^denote statistically significant values over MDA-MB231-adipocytes co-culture (^#^*p* < 0.05). (**B**) Confluent monolayers of MDA-MB231 were wounded by manually scratching and incubated with CM collected from human adipocytes for 24 h in presence of 6 μg/ml CCL5 Ab, 5 μg/ml CCR5 pep or 10 μM AG1024. Images of wound gap were taken at 0 and 24 h by a digital camera coupled to the microscope and percentage of wound distance was calculated with the camera software. The results have been reported as percentage of gap closure at 24 h compared with time 0. A complete gap closure was considered as 100%. *denote statistically significant values over control (***p < 0.001). ^#^denote statistically significant values over Adipo-CM treated cells (^#^*p* < 0.05). The pictures are representative of wound gaps at 0 point and upon 24 h of scratch assay. Data in the graphs represent the mean ± SD of at least three independent triplicate experiments.

We have previously shown that glucose increases the release of CCL5 and IGF-1 by adipocytes [11]. Now, we provide evidences that glucose did not directly interfere with their secretion by cancer cells (Supplementary Figure 2A–2B). In order to address the biological relevance of CCL5 and IGF-1 as adipocyte-derived motility promoting factors, MDA-MB231 were co-cultured with human adipocytes in presence of specific inhibitors of CCL5 action: a monoclonal antibody raised against CCL5 (CCL5-Ab) or a peptide for the CCL5 receptor CCR5 (CCR5-pep) [13, 14]. Both CCL5-Ab and CCR5-pep almost completely prevented STAT3 phosphorylation (Supplementary Figure 3) as well as the effect of adipocytes on MDA-MB231 invasiveness (Figure 2A). At variance, AG1024, a tyrosine kinase inhibitor of IGF-1 receptor [15], did not significantly reduce adipocyte action (Figure 2A). Similarly, CCL5 inhibition led to a significant reduction of wound closure of CM-treated MDA-MB231 (Figure 2B). Conversely, IGF-1 receptor inhibition did not interfere with adipocyte-induced cancer cell motility (Figure 2B).

**Figure 3:**
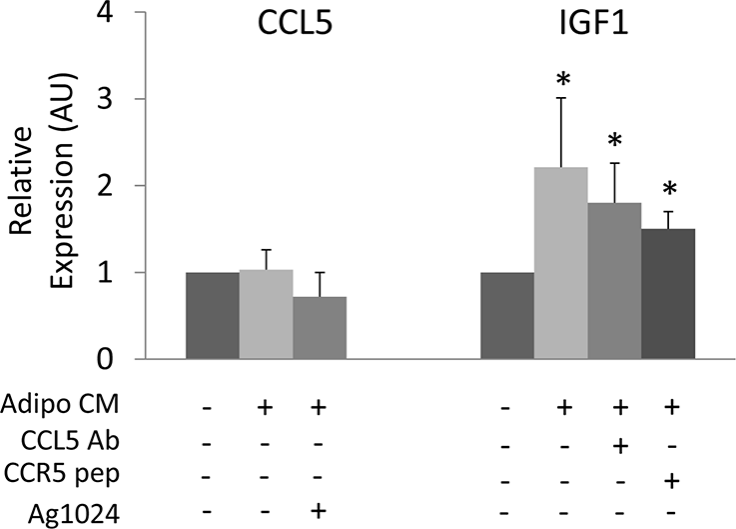
Cross-regulation of CCL5 and IGF-1 in cancer cells MDA-MB231 were incubated with CM collected from human adipocytes for 24 h in presence or absence of 6 μg/ml CCL5 Ab, 5 μg/ml CCR5 pep or 10 μM AG1024. Next, mRNA levels of human CCL5 and IGF-1 were determined by real-time RT-PCR analysis as described in Materials and Methods. Bars represent the mean ± SD of four independent experiments. Bars show mRNA levels in these cells relative to those in untreated cells (cells in serum-free medium). Asterisks denote statistically significant values over untreated cells (*p < 0.05).

Next, we addressed whether adipocytes may control CCL5 and IGF-1 production by cancer cells. To this aim, we have measured CCL5 and IGF-1 mRNA levels in MDA-MB231 cells following adipocyte CM exposure. Adipocyte CM did not induce any significant increase of CCL5 mRNA levels in MDA-MB231 cells, while inducing IGF-1 expression (Figure 3). The presence of CCL5-Ab or CCR5-pep did not reduce CM-induced expression of IGF-1, suggesting that adipocyte-released factors different from CCL5 may control IGF-1 production by cancer cells. Moreover, AG1024 treatment did not modify CCL5 expression in MDA-MB231 cells thus indicating that IGF-1 did not control CCL5 in cancer cells (Figure 3).

### CCL5 and IGF-1 detection in peritumoral adipose tissue of women with TNBC

Next, we evaluated whether CCL5 and IGF-1 could be detected in peritumoral adipose tissue of women with breast cancer. We selected 40 invasive ductal TNBC samples. Patients’ mean age was 59 years (range from 27 to 93); 12 patients (30%) were pre-menopausal and 28 (70%) post-menopausal. Tumors larger than 2 cm occurred in 52.5% (21/40) of patients. 95% (38/40) of patients had tumor with poorly differentiated cells (grade 3), while only 5% (2/40) had tumor with moderately differentiated cells (grade 2). None of the tumors was of grade 1. Metastatic lymph nodes were found in 40% (16/40) of patients at surgery and 30% (12/40) of patients developed distant metastases. The expression of the proliferation factor Ki67 was high (> 30%) in 55% (22/40), and low (≤ 30%) in 45% (18/40) of specimens. 17.5% (7/32) of patients were diabetic and 32.5% (13/31) had a BMI greater than 30 (Table 1).

**Table 1:** Clinical and pathological features of TNBC samples

	Patients (*n* = 40)
***Age***	
**Mean:59 y (range: 27–93)**	
**< 40**	4 (10%)
**≥ 40 ≤ 60**	17 (42.5%)
**> 60**	19 (47.5%)
***Grade***	
**I**	0 (0%)
**II**	2 (5%)
**III**	38 (95%)
***Size (T)***	
**≤ 2 cm**	20 (50%)
**> 2 ≤ 5 cm**	17 (42.5%)
**> 5**	3 (3.5%)
***LNM***	
**Yes**	16 (40%)
**No**	24 (60%)
***Metastasis***	
**Yes**	12 (30%)
**No**	28 (70%)
***Ki-67***	
**> 30**	22 (55%)
**≤ 30**	18 (45%)
***BMI***	
**Mean: 27.65 (range: 20.03–37.92)**	
**< 30**	18 (45%)
**≥ 30**	13 (32,5%)
**Unknown**	9 (22.5%)
***Diabetes***	
**Yes**	8 (20%)
**No**	24 (60%)
**Unknown**	8 (20%)
***Follow up***	
**NED**	16 (40%)
**AWD**	10 (25%)
**DOD**	3 (7.5%)
**Unknown**	11 (27.5%)

LNM = Limph node metastasis; NED = No evidence of disease; AWD = Alive with disease; DOD = Dead of disease.

**Figure 4:**
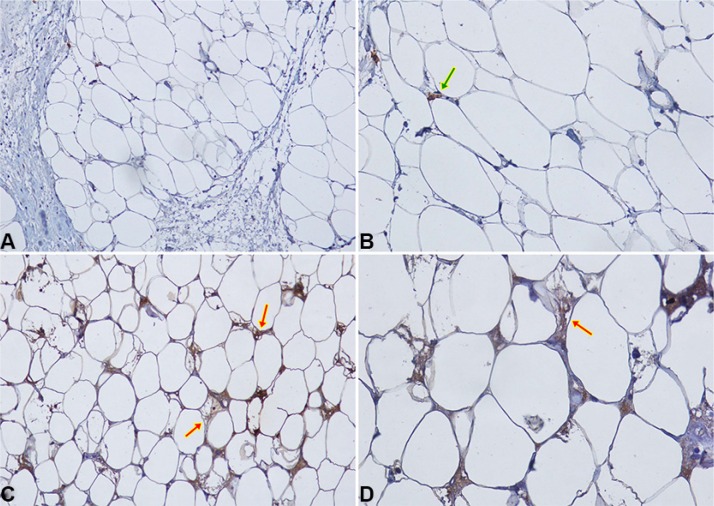
CCL5 IHC staining in peritumoral adipocytes of TNBC samples (**A–B**) Negative expression of CCL5 in peritumoral adipocytes of a TNBC sample at 20× (A) and 40× (B) magnification. Macrophage and lymphocyte CCL5 staining (green arrow) represents positive internal controls; (**C–D**) An example of specific CCL5 staining in peritumoral adipocytes (red arrows) is shown at 20× (C) and 40× (D) magnification.

**Figure 5:**
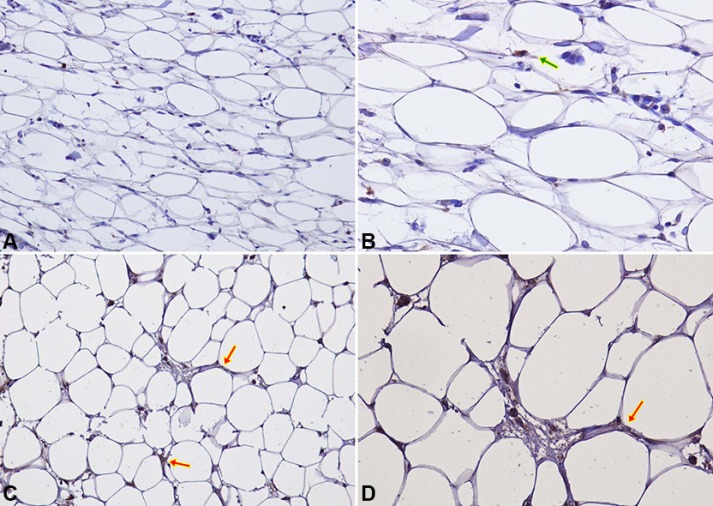
IGF-1 IHC staining in peritumoral adipocytes of TNBC samples (**A–B**) Negative expression of IGF-1 in peritumoral adipocytes of a TNBC sample at 20× (A) and 40× (B) magnification. Macrophage and lymphocyte IGF-1 staining (green arrow) represents positive internal controls; (**C–D**) An example of specific IGF-1 staining in peritumoral adipocytes (red arrows) is shown at 20× (C) and 40× (D) magnification.

CCL5 and IGF-1 specific staining was detected in peritumoral adipocytes of TNBC samples (up to 1 cm distance from the tumour). CCL5 staining in peritumoral adipose tissue was detected in 16/40 samples (40%) (Figure 4). Adipose tissue IGF-1 staining was detected in 20/40 samples (50%) (Figure 5).

### Peritumoral adipose tissue CCL5 staining correlates with lymph node and distant metastases

Statistical analysis showed that CCL5 staining in peritumoral adipocytes of TNBC samples was not associated with patient age, menopausal status, BMI, diagnosis of diabetes, tumor histotype, size and grading (Table 2). CCL5 protein staining showed a positive, although not statistically significant correlation trend with Ki67 proliferation index (*P*-value = 0.085). Interestingly, CCL5 staining in peritumoral adipocyte was significantly associated with lymph node (*P*-value = 0.05) and distant metastases (*P*-value = 0.001) (Table 2). A positive trend (*P*-value = 0.095) between CCL5 immunoreactivity and fasting glucose levels was also revealed. No significant correlation was observed for IGF-1 staining in the adipocytes of TNBC samples with age, menopausal status, BMI, diabetes, glycaemic levels, tumour histotype, size and grading, Ki67 expression, lymph node and distant metastases (Table 2). CCL5 and IGF-1 staining was detected in peritumoral adipose tissue of 25 samples of ER positive patients whose clinical and pathological features have been described in Supplementary Table 1. CCL5 was detected in 19/25 samples (76%), while IGF-1 in 22/25 samples (88%) (data not shown). No statistically significant association was found between CCL5 or IGF-1 staining and clinical pathological parameters in ER positive cases (Supplementary Table 1).

**Table 2:** Association between CCL5 and IGF1 staining in peritumoral adipocytes and clinical pathological features

	CCL5				IGF1			
	Negative	Positive	*P* value	R pearson	Negative	Positive	*P* value	R pearson
***Age (n = 40)***								
**< 40**	4 (100%)	0 (0%)	0.247	0.253	2 (50%)	2 (50%)	0.785	−0.66
**≥ 40 ≤ 60**	12 (70.6%)	5 (29.4%)			7 (41.2%)	10 (58.8%)		
**> 60**	11 (57.9%)	8 (42.1%)			10 (52.6%)	9 (47.4%)		
***Menopause (n = 40)***								
**Pre-**	8 (66.6%)	4 (43.4%)	0,941	−0,012	5 (41.7%)	7 (58.3%)	0,490	−0.109
**Post-**	19 (67.9%)	9 (32.1%)			15 (53.6%)	13 (46.4%)		
***Size (n = 40)***								
**≤ 2 cm**	14 (70%)	6 (30%)	0.943	0.045	9 (45%)	11 (55%)	0.781	−0.086
**> 2 ≤ 5**	11 (64.7%)	6 (35.3%)			8 (47%)	9 (53%)		
**> 5**	2 (66.6%)	1 (33.4%)			2 (66.6%)	1 (33.4%)		
***LNM (n = 40)***								
**Negative**	19 (82.6%)	5 (17.4%)	0.05*	**0.305**	10 (41.6%)	14 (58.4%)	0.366	−0.143
**Positive**	8 (50%)	8 (50%)			9 (56.3%)	7 (43.7%)		
***Metastasis (n = 40)***								
**Negative**	24 (82.6%)	5 (17.4%)	0.001**	**0,529**	12 (41.4%)	17 (58.6%)	0.208	−0.199
**Positive**	3 (27.3%)	8 (32.7%)			7 (63.6%)	4 (36.4%)		
***Grade (n = 40)***								
**G1**	0 (0%)	0 (0%)	0.314	0.159	0 (0%)	0 (0%)	0.942	0.011
**G2**	2 (100%)	0 (0%)			1 (50%)	1 (50%)		
**G3**	25 (65.8%)	13 (34.2%)			18 (47.4%)	20 (52.6%)		
***Ki67 (n = 40)***								
**≤ 30%**	14 (82.3%)	3 (17.7%)	*0.085*	*0.273*	7 (41.2%)	10 (58.8%)	0.491	−0.109
**> 30%**	13 (56.5%)	10 (43.5%)			12 (52.3%)	11 (47.7%)		
***BMI (n = 31)***								
**< 30**	13 (72.2%)	5 (37.8%)	0.530	0.113	7 (38.9%)	11 (61.1%)	0.686	−0.073
**≥ 30**	8 (61.5%)	5 (38.5%)			6 (46.2%)	7 (53.8%)		
***Diabetes (n = 32)***								
**No**	17 (70.8%)	7 (29.2%)	0.283	0.190	12 (50%)	12 (50%)	0.539	0.108
**Yes**	4 (50%)	4 (50%)			3 (37.5%)	5 (62.5%)		
***Glycaemia (n = 22)***								
**< 110**	12 (75%)	4 (25%)	*0.095*	*0.102*	6 (37.5%)	10 (62.5%)	0.479	−0.044
**≥ 110 < 126**	0 (0%)	2 (100%)			0 (0%)	2 (100%)		
**≥ 126**	3 (75%)	1 (25%)			2 (50%)	2 (50%)		

LNM = Limph Node Metastasis. Fasting glucose levels were determined one week before the surgery. * denote statistically significant values (*p < 0.05 **p < 0.01).

**Figure 6:**
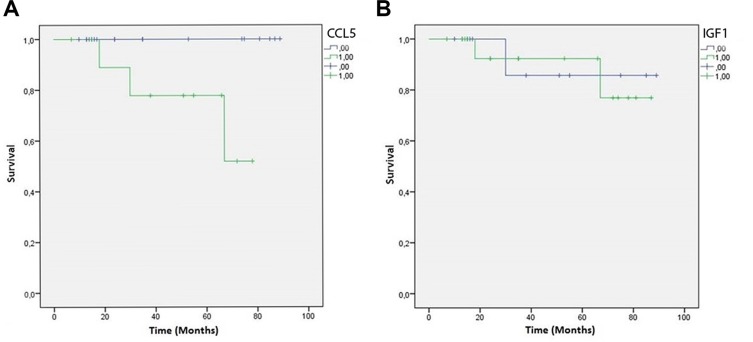
CCL5 and IGF-1 Kaplan-Meier overall survival curves Green lines represent patients expressing CCL5 (**A**) or IGF-1 (**B**) in peritumoral adipose tissue. Blue lines represent patients who do not express CCL5 (a) or IGF-1 (b) in peritumoral adipose tissue.

Finally, Kaplan-Meier curves showed a significant negative correlation between CCL5 immunoreactivity in the peritumoral adipose tissue of TNBC samples and patient overall survival (*P*-value = 0.039) (Figure 6A). At variance, IGF-1 peritumoral adipocyte staining was not associated with the overall survival (*P*-value = 0.909) (Figure 6B).

## DISCUSSION

Interactions between tumor cells and the associated stroma may promote disease progression and worsen prognosis [16]. In cancer, the coordinated intercellular interactions are disrupted as the tumor acquires the capacity to chronically circumvent normalizing cues from the microenvironment, and in turn, the microenvironment evolves to accommodate the growing tumor [17].

We have addressed whether peritumoral adipose tissue may modify cancer cell invasiveness. For this purpose, we have used Triple-Negative Breast Cancer (TNBC) cells (which stain negative for estrogen receptor, progesterone receptor, and HER-2) and human mammary adipocytes. TNBCs account for 10–24% of invasive breast cancers and are typically high-grade tumors with different histological types. Usually, patients with TNBC have a higher recurrence rate after diagnosis, a short disease-free interval and a reduced overall survival, mainly for the lack of targeted therapies [18].

Adipocytes are active and important modulators of mammary tumor microenvironment [19]. Data mainly obtained in cultured murine cell models have suggested that adipocyte factors, including a wide array of adipokines, cytokines and growth factors, promote survival, growth and motility of cancer cells [5, 11, 20].

Here, we show that TNBC cells display increased motility and invasiveness when co-cultured with human adipocytes. Interestingly, raising glucose concentrations further increased the promoting action of adipocytes on cancer cell motility. As also previously reported by others, higher glucose concentrations *per se* may enhance breast cancer cell growth and motility, by providing fuel for the increasing energy demand [21]. However, experiments with co-culture and conditioned media have indicated that glucose increases the release of adipocyte factors, which, even in lower glucose concentrations, are capable to enhance cancer cell motility and invasiveness. Thus, high glucose levels may facilitate cancer progression by acting either directly on cancer cells or indirectly on the surrounding adipose cells.

These *in vitro* observations are consistent with the evidence that: i) the epidemic surge of obesity and diabetes mellitus is paralleled by increased onset and progression of oncologic disorders, including breast cancer [1, 22]; ii) hyperglycaemia represents an independent risk factor for cancer progression [23, 24]; iii) tumour microenvironment responds to metabolic imbalance and accounts for several modifications occurring in cancer phenotypes [12, 25, 26].

All together these observations indicate that the adipose tissue within the tumour microenvironment may sense metabolic derangements and worsen breast cancer progression.

When cultured in high glucose concentrations or with fatty acids, adipocytes release a larger amount of IGF-1 and CCL5, without changing the secretion of several other factors [11]. We have now shown that CCL5 is needed for breast cancer cell motility and invasion promoted by adipocytes. Indeed, either blocking CCL5 with a neutralizing antibody or preventing CCL5 binding to its CCR5 receptor reduced adipocyte effect on TNBC cell motility and invasiveness. At variance, an IGF-1/Insulin pathway inhibitor had no effect on adipocyte action, suggesting that adipocyte-released IGF-1 is dispensable (notably, insulin is not released by adipocytes and is not present in culture medium).

CCL5 is a chemokine that has been associated to several forms of cancer [27]. Elevated circulating levels of CCL5 have been found both in diabetic and in obese individuals [28, 29]. Nevertheless, CCL5/CCR5 expression levels are different among the different genetic subtypes of breast cancer [30–33] and may represent a negative prognostic factor [34, 35]. However, tumor-derived CCL5 apparently does not contribute to breast cancer progression [36]. It has been shown that CCL5 induces the invasion of basal breast cancer cells (i.e. MDA-MB-231 cells) but not of luminal breast cancer cells (i.e. MCF-7 cells) [33, 37]. In agreement, increased CCL5 circulating levels have been found predominantly in ER-negative patients [38].

Here, we have developed an immunohistochemical method to detect CCL5 and IGF-1 in tumor-associated adipose tissue specimens. This was a relevant achievement since very few markers have been analyzed in adipose cells through in-situ techniques [39–41]. We were also able to establish an adequate evaluation score. Interestingly, in TNBCs, CCL5, while not IGF-1, immunodetection is significantly associated with lymph node and distant metastases. Consistently, a negative association of CCL5 staining in TNBC-associated adipose tissue with overall survival is suggestive of a more aggressive cancer behavior. At the best of our knowledge, this is the first observation of a potential prognostic marker in peri-tumoral adipose tissue. Further work is needed to analyze the association between CCL5 and IGF-1 staining in adipose tissue and cancer features of patient with ER positive cancer.

Most likely, CCL5 is largely produced by adipocytes. Indeed, no induction of CCL5 gene expression by adipocyte factors has been detected in breast cancer cells. Since CCL5 could act locally promoting cell movement, it could be involved in cancer invasion of the surrounding adipose tissue, which is one of the biologic indicators of tumor aggressiveness [42].

CCL5 binds to its cognate receptor CCR5 and activates Jak kinases, STAT3, mTOR and p38 MAP kinase regulating the engagement of multiple signaling pathways [13, 43, 44]. It mediates the trafficking and homing of classical lymphoid cells such as T cells and monocytes, but also acts on a range of other cells, including basophils, eosinophils, natural killer cells, dendritic cells and mast cells [45]. Upon recruitment of immune cells, inflamed adipose tissue may further promote growth of malignant cells by inducing vascular endothelium and activating proinflammatory cells [46].

Recently, genomic profiling studies have identified specific subtypes for TNBC with differential aggressiveness potential [47, 48]. These studies have evidenced that the Basal-Like Immune Activated subtype, which display up-regulation of genes controlling B cell, T cell, natural killer cell functions and inflammatory cytokines, was among the most aggressive molecular subtypes. Therefore, it is plausible that excess adiposity, as well as high blood glucose levels, may contribute to the production of inflammation/immunity-related factors, which may worsen cancer prognosis in metabolically deranged individuals. It should be pointed out that, although not reaching statistical significance, higher glycaemic levels are also associated with CCL5 detection in TNBC specimens. Further studies are needed to analyse CCL5 levels in serum of TNBC patients.

Thus, we described that CCL5 release by adipocytes contributes to increase motility and invasiveness of breast cancer cells. CCL5 is detectable in peritumoral adipose tissue of TNBCs and correlates with lymph node and distant metastases and with the reduction of patient overall survival. This is a relevant issue since, so far, no effective molecular targeted drug is available for TNBC. In this regard, CCR5 antagonists are widely used for the treatment of HIV infection [49]. Moreover, in animal models, CCL5 knockout does not affect general physiology and broad immunity [50], suggesting that specific targeted inhibition may have strong therapeutic impact without over-toxicity.

## MATERIALS AND METHODS

### Materials

Media, sera, and antibiotics for cell culture were from Lonza (Lonza Group Ltd, Basel, Switzerland). Anti-CCL5 and IGF1 antibodies were from Abcam (Cambridge, UK). Anti-Erα, PR, c-Erb B2 and Ki67 antibodies were purchased from DAKO (Ely, UK). All the other chemicals were from Sigma-Aldrich (St. Louis, MO, USA).

### Peptide synthesis

The synthesis of peptide CCR5-pep (sequence: AFDWTFVPSLIL-NH_2_) was performed according to the solid-phase approach using standard Fmoc methodology in a manual reaction vessel. Nα-Fmoc-protected amino acids, Rinkamide-resin, N-hydroxy-benzotriazole (HOBt), 2-(1Hbenzotriazole-1-yl)21,1,3,3-tetramethyluronium hexafluorophosphate (HBTU), N, N-diisopropylethyl-amine (DIPEA), piperidine and trifluoroacetic acid were purchased from Iris Biotech (Germany). Peptide synthesis solvents, reagents, as well as CH_3_CN for high performance liquid chromatography (HPLC) were reagent grade and were acquired from commercial sources and used without further purification unless otherwise noted. The first amino acid, NαFmoc-Leu-OH was linked on to the Rink resin (100–200 mesh, 1% divinylbenzene, 0.75 mmol/g) previously deprotected by a 25% piperidine solution in N, N-dimethylformamide (DMF) for 30 min.

The following protected amino acids were then added stepwise: Nα-Fmoc-Ile-OH, Nα-Fmoc-Leu-OH, Nα-Fmoc-Ser(tBU)-OH, Nα-Fmoc-Pro-OH, Nα-Fmoc-Val-OH, Nα-Fmoc-Phe-OH, Nα-Fmoc-Thr(tBU)-OH, Nα-Fmoc-Trp(Boc)-OH, Nα-Fmoc-Asp(OtBU)-OH, Nα-Fmoc-Ala-OH,. Each coupling reaction was accomplished using a threefold excess of amino acid with HBTU and HOBt in the presence of DIPEA (6 eq.). The Na-Fmoc protecting groups were removed by treating the protected peptide resin with a 25% solution of piperidine in DMF (1 × 5 min and 1 × 25 min).

The peptide resin was washed three times with DMF, and the next coupling step was initiated in a stepwise manner. The peptide resin was washed with dichloromethane (DCM) (3×), DMF (3×), and DCM (3×), and the deprotection protocol was repeated after each coupling step. In addition, after each step of deprotection and after each coupling step, Kaiser test was performed to confirm the complete removal of the Fmoc protecting group, respectively, and to verify that complete coupling has occurred on all the free amines on the resin.

The N-terminal Fmoc group was removed as described above, and the peptide was released from the resin with trifluoroacetic acid (TFA)/triisopropylsilane(iPr_3_SiH)/H2O (90:5:5) for 3 h. The resin was removed by filtration, and the crude peptide was recovered by precipitation with cold anhydrous ethyl ether to give a white powder and then lyophilized.

### Purification and characterization

All crude peptides were purified by reversed-phase high performance liquid chromatography (RP-HPLC) on a semipreparative C18-bonded silica column (Phenomenex, Jupiter, 250 mm 3 10 mm) using a Shimadzu SPD 10A UV/VIS detector, with detection at 210 and 254 nm.

The column was perfused at a flow rate of 3 ml/min for 40 min with solvent A (10%, vol/vol, water in 0.1% aqueous TFA), and a linear gradient from 10 to 90% of solvent B (80%, vol/vol, acetonitrile in 0.1% aqueous TFA). Analytical purity and retention time (tR) of each peptide were determined using HPLC conditions in the above solvent system (solvents A and B) programmed at a flow rate of 1 ml/min using a linear gradient from 10 to 90% B over 25 min, fitted with C-18 column Phenomenex, Juppiter C-18 column (250 mm 3 4.60 mm; 5 mm).

All analogues showed **>** 97% purity when monitored at 215 nm. Homogeneous fractions, as established using analytical HPLC, were pooled and lyophilized.

Peptides molecular weights were determined by electrospray ionization mass spectrometry (ESI-MS). ESI-MS analysis in positive ion mode were made using a Finnigan liquid chromatography quadrupole mass spectrometry (LCQ) ion trap instrument, manufactured by Thermo Finnigan (San Jose, CA), equipped with the Excalibur software for processing the data acquired. The sample was dissolved in a mixture of water and methanol (50/50) and injected directly into the electrospray source, using a syringe pump, which maintains constant flow at 5 ml/min. The temperature of the capillary was set at 220°C.

### Cell cultures

MDA-MB-231 (ERα, PgR and HER2 negative) and MCF-7 (ERα, PgR positive and HER2 negative) human breast cancer cells were cultured in Dulbecco's modified Eagle's medium (DMEM) supplemented with 10% fetal bovine serum (FBS) and 2 mM glutamine, 100 IU/ml penicillin, 100 IU/ml streptomycin. Cultures were kept in humidified atmosphere of 95% air and 5% CO_2_ at 37 C. Human adipose tissue samples were obtained from mammary adipose tissue biopsies of healthy women (*n* = 12; age 25–63 years; BMI 24.2–29.0) undergoing surgical mammary reduction. All women were otherwise healthy and free of metabolic or endocrine diseases. Informed consent was obtained from every subject before the surgical procedure. The ethical committee of the University of Naples approved this procedure. Adipose tissue was digested with collagenase and Adipose-derived Stromal Vascular Fraction cells (SVF) were isolated and differentiated as previously reported [51]. Conditioned media (CM) were obtained by incubating the cells for 8 h with serum-free DMEM 0.25% Bovine Serum Albumin (BSA) after two washes with Phosphate Buffered Saline (PBS). After the incubation, medium was collected and centrifuged at 14000 *g* to remove cellular debris and placed onto recipient cells.

### Co-culture and invasion assay

Cell invasiveness was examined using a reconstituted extracellular matrix (1.5 mg/ml Matrigel; BD Biosciences, Bedford, MA, USA) coated on polycarbonate membranes of the upper compartment of a 24 well transwell system (8 μm pore size, Costar, Cambridge, MA, USA). 24 h serum-starved MDA-MB231 cells (1 × 10^3^ cells/well) were seeded on the matrigel-coated membrane and co-cultivated with either differentiated adipocytes or with undifferentiated SVF cells in the bottom chamber in serum-free medium. Alternatively, adipocyte CM was placed in the bottom of the transwell. Then, cells were allowed to invade the matrix and migrate into the lower chamber at 37 C in a 5% CO_2_ atmosphere saturated with H_2_O for 24 h. At the end of incubation, the upper surface of the membrane was swiped to remove the attached cells. The cells that had migrated to the lower side of the filter were fixed with 11% glutaraldehyde for 15 min at room temperature, washed three times with PBS, and stained with 0.1% crystal violet-20% methanol for 20 min at room temperature. After three PBS washes and complete drying at room temperature, the crystal violet was solubilized by immersing the filters in 10% acetic acid. The concentration of the solubilized crystal violet was evaluated as absorbance at 540 nm.

### Scratch assay

Scratch assay was performed as previously described [52]. Briefly, MDA-MB231 cells were starved in serum-free DMEM-F12 0.25% BSA for 16 h. Confluent monolayers were wounded by manually scratching with a p20 pipette tip. They were next washed twice with PBS and incubated at 37 C with mitomycin C (10 mg/ml; Sigma Aldrich, St. Louis, MO, USA) and adipocyte CM. Images of wound gap were taken at 0 and 24 h by the Olympus DP20 microscope digital camera system (Olympus Corporation, Tokyo, Japan) and percentage of closure was calculated with a dedicated software.

### Real-time RT-PCR analysis

Total RNA was isolated from MDA-MB231 cells by using the Rneasy Kit (Qiagen, Valencia, CA, USA) according to the manufacturer's instruction. 1 μg RNA was reverse transcribed using SuperScript III Reverse Transcriptase (Life Technologies, Carlsbad, CA, USA). Quantitative real-time RT-PCR was performed with SYBR Green mix (Bio-Rad, Hercules, CA, USA) using an iCycler IQ multicolor Real-Time PCR Detection System (Bio-Rad, Hercules, CA, USA). All reactions were performed in triplicate and β-actin was used as an internal standard. Primer sequences are described in Table 3.

**Table 3:** Primer sequences used in real-time RT-PCR analysis

**IGF-1 primers**	Forward 5′-GCA GAA CCT GTT TGG CTC TC-3′ Reverse 5′-TAT GGT CTT TGC AAG GGA GG-3′
**CCL5 primers**	Forward 5′-CAG CAC GTG GAC CTC GCA CA-3′ Reverse 5′-GGCAGTGGGCGGGCAATGTA-3′
**β-actin primers**	Forward 5′-GCGTGACATCAAAGAGAAG-3′ Reverse 5′-ACTGTGTTGGCATAGAGG-3′

### Patients and specimens

40 patients, who were diagnosed as Triple Negative Breast Cancer (TNBC) were enrolled in this study among those who underwent breast surgery from 2003 to 2010 at the National Cancer Institute “Giovanni Pascale Foundation” of Naples, Italy. These patients were different from those ones enrolled for adipose tissue biopsies. As for Institutional statistics, TNBCs represent 15–19% of the total number of breast cancer surgical samples. All cases of TNBC samples were reviewed according to WHO classification criteria, using standard tissue sections and appropriate immunohistochemical slides. Medical records for all cases of TNBC samples were reviewed for clinical information, including histologic parameters that were determined from the H and E slides. The following clinical and pathological parameters were evaluated for each tumor included in the study: patient age at initial diagnosis, tumor size, histologic subtype, nuclear grade, nodal status, number of positive lymph nodes, tumor stage, tumor recurrence or distant metastasis and type of surgery (for tumor removal). In addition, all specimens were characterized for all routinely diagnostic immunophenotypic parameters.

### Immunohistochemistry analysis

CCL5, IGF-1, ERα, PR, c-ErbB and KI67 staining was evaluated on slides of formalin-fixed, paraffin-embedded tissues by immunohistochemical staining. Paraffin slides were deparaffinized in xylene and rehydrated through graded alcohols. Antigen retrieval was performed on slides heated in 0.01 M citrate buffer (pH 6.0) in a bath for 20 minutes at 97 C. After antigen retrieval, the slides were allowed to cool. The slides were rinsed with Tris Buffered Saline (TBS) and the endogenous peroxidase was inactivated with 3% hydrogen peroxide. After protein blocking (BSA 5% PBS 1×), slides were incubated with primary antibody to human CCL5 (dilution 1:100) and IGF-1 (dilution 1:500) over night and to human ERα (dilution 1:35), PR (dilution 1:50), c-Erb B2 (dilution 1:300) and Ki67 (dilution 1:75) for 30 min. The sections were rinsed in TBS and incubated for 20 min with Biotinylated Secondary Antibody (RE7103, Novocastra, Nussloch, Germany). Then, sections were rinsed in TBS and incubated for 20 min with Streptavidin-HRP (RE7104, Novocastra, Nussloch, Germany). Peroxidase reactivity was visualized using a 3, 3′-diaminobenzidine (DAB). Finally, the sections were counterstained with hematoxylin and mounted.

### Immunohistochemistry evaluation

Two trained pathologists using a light microscopy evaluated antigen immunoreactivity independently. Observer was unaware of the clinical outcome. For each sample, at least five High Power Fields (HPF) (inside the tumor and in the area exhibiting tumor invasion) and > 500 cells were analysed. Using a microscopically semi-quantitative scoring system and referring to each antigen scoring method in other studies, we evaluated the intensity, extent and subcellular distribution of CCL5 and IGF-1.

The proliferative index Ki67 was defined as the percentage of immunoreactive tumour cells out of the total number of cells. The percentage of positive cells per case was scored according to 2 different groups: group 1: < 30% (low proliferative activity); group 2: > 30% (high proliferative activity).

There were no standardized criteria for CCL5 and IGF-1 adipose staining evaluation. We evaluated CCL5 and IGF1 protein reactivity in peritumoral adipocytes (until one centimeter from the tumor), considering only the positive or negative staining. CCL5 and IGF-1 detection in lymphocytes and macrophages was considered as internal positive control.

Other routinely used diagnostic markers (ERα, PR and c-Erb B2) were evaluated as previously described [53].

### Statistical analysis

The Pearson χ^2^ test was used to analyze the association between CCL5 and IGF-1 staining in peritumoral adipocytes and clinical pathological features included in the study. The level of significance was defined as *P* < 0.05. Overall survival (OS) curves were calculated using the Kaplan-Meier method and the significance was evaluated using the Mantel-Cox log-rank test. OS was defined as the time from diagnosis (first biopsy) to death by any cause or until the most recent follow-up. The follow-up duration was five years. All the statistical analyses were carried out using the Statistical Package for Social Science v. 20 software (SPSS Inc., Chicago, IL, USA).

## SUPPLEMENTARY MATERIALS FIGURES AND TABLE


