# Supplementary material for: Adipose microenvironment promotes triple negative breast cancer cell invasiveness and dissemination by producing CCL5
Source: Oncotarget. 2016 Mar 24;7(17):24495–509. doi: 10.18632/oncotarget.8336 (PMC5029717; doi:10.18632/oncotarget.8336)
Supplement: Supplementary file 1 [file oncotarget-07-24495-s001.pdf]

Adipose microenvironment promotes triple negative breast cancer cell invasiveness and dissemination by producing CCL5

Supplementary Materials

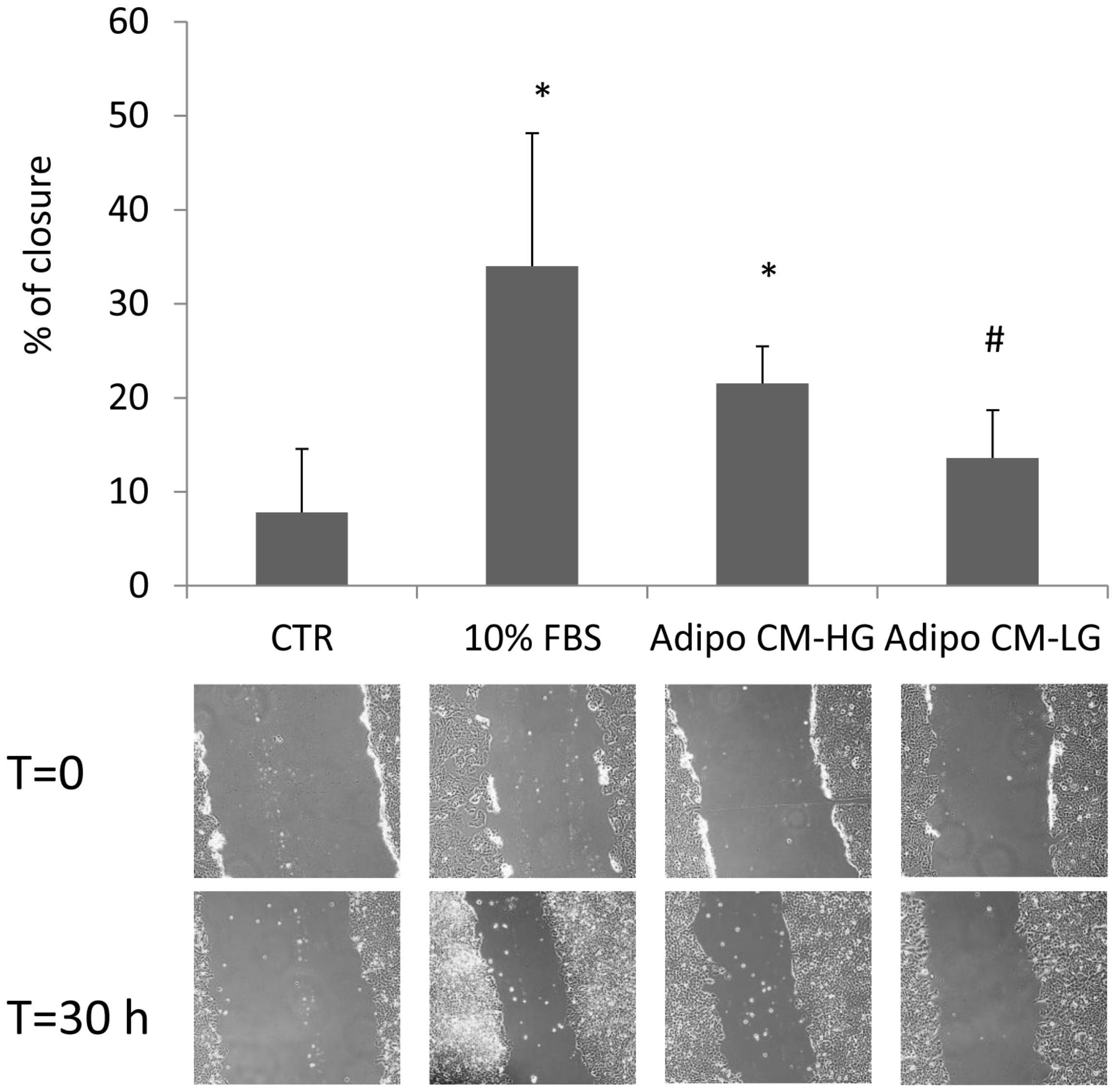

Supplementary Figure S1: Effect of glucose-treated adipocytes on ER-a+ breast cancer cell motility. Confluent monolayers of MCF-7 were wounded by manually scratching and incubated with CM collected from human adipocytes pre-incubated with either LG or HG medium for 24 h and further incubated with regular glucose serum-free medium for 8 h. Images of wound gap were taken at 0 and 30 h by a digital camera coupled to the microscope and percentage of wound distance was calculated with the camera software. The results have been reported as percentage of gap closure at 24 h compared with time 0. A complete gap closure was considered as 100%. *denote statistically significant values over MCF-7 cells incubated in regular glucose serum-free medium (*$p < 0.05$). #denote statistically significant values of CM-HG over CM-LG (#$p < 0.05$). The pictures are representative of wound gaps at 0 point and upon 30 h of scratch assay.

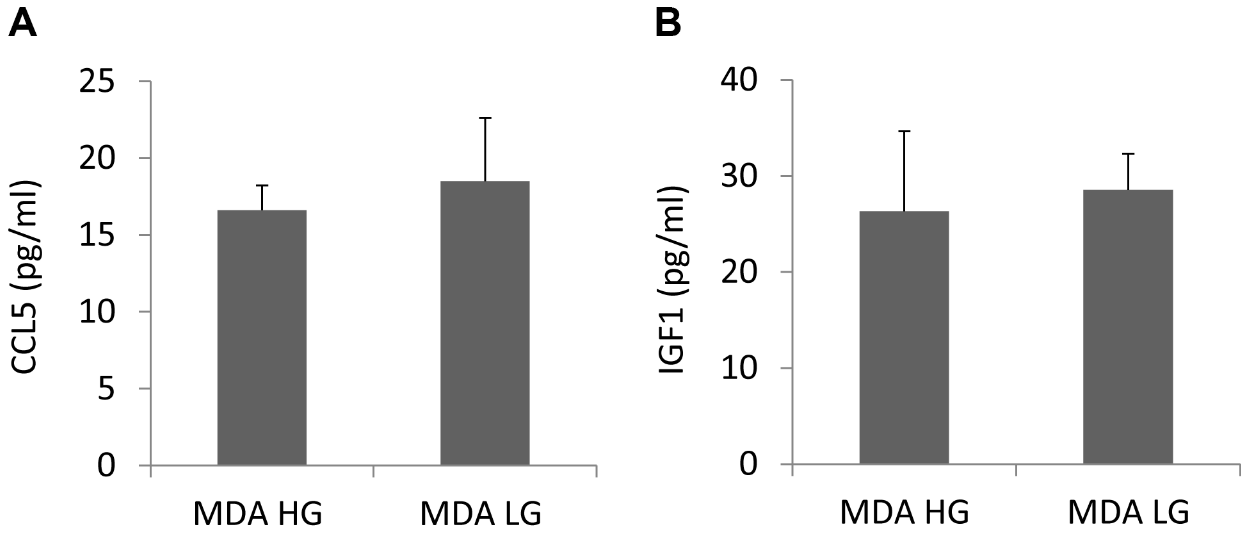

Supplementary Figure S2: Effect of glucose on MDA-MB231 cell release of CCL5 and IGF1. MDA-MB231 cells were incubated with HG medium or with LG medium for 24 h. Then, they were incubated with serum-free medium for 8 h. CCL5 (**A**) and IGF1 (**B**) concentrations were determined by using Quantikine ELISA kits (R & D System, Minneapolis, MN, USA) according to manufacturer's instructions.

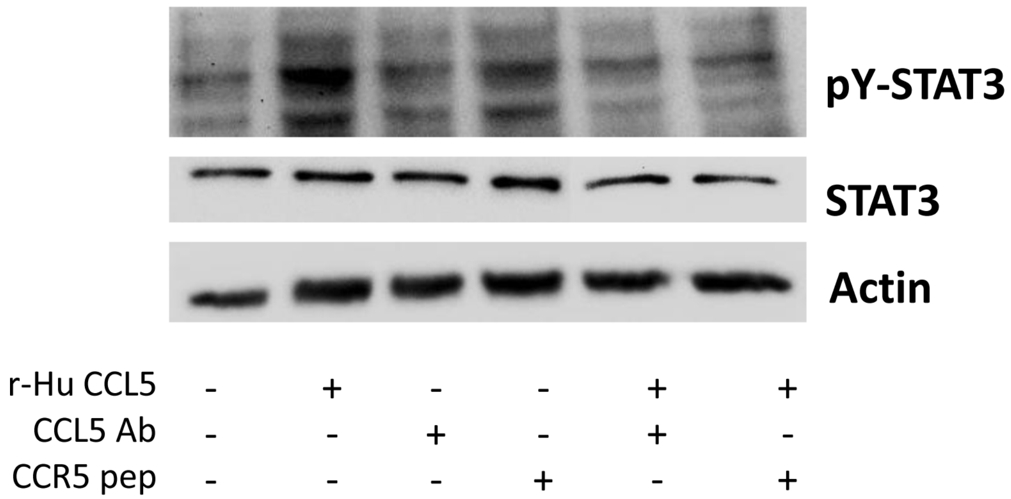

Supplementary Figure S3: Effect of CCL5 inhibitors on CCL5 pathway. MDA MB-231 cells were serum starved for 16 h and next exposed to 25 ng/ml recombinant human CCL5 (PeproTech (Rocky Hill, NJ, USA), 6 |ig/ml CCL5 Ab or 5 |ig/ml CCR5 peptide for 1 h. Cells were solubilized for 20 min at 4 C with lysis buffer containing 50 mM HEPES, 150 mM NaCI, 10 mM EDTA, 10 mM l\ la4P207, 2 mM sodium orthovanadate, 50 mM NaF, 1 mM phenylmethylsulfonyl fluoride,10 |ig/ml aprotinin, 10 |ig/ml leupeptin, pH 7.4, and 1% (v/v) Triton X-100. Lysates were clarified by centrifugation at 12,000g for 20 min at 4 C. 20 |ig protein/sample were separated by SDS-polyacrylamide gel electrophoresis and blotted on Immobilon-P membranes (Millipore, Billerica, MA, USA). Membranes were blocked for 1 h in TBS (10 mM Tris-HCI, pH 7.4, and 140 mM NaCI) containing 3% (w/v) BSA and then incubated with phospho- Tyr705 Signal Transducer and Activator of Transcription (STAT) 3 antibodies (Cell Signaling Technology, Danvers, MA, USA). Then, membranes were blotted with STAT3 and actin antibodies (Santa Cruz Biotechnology, Santa Cruz, CA, USA). The filters were revealed by ECL and autoradiography.

Supplementary Table S1: Association between CCL5 and IGF1 staining in peritumoral adipocytes and clinical pathological features of ER positive cases

	CCL5				IGF1			
	**Negative**	**Positive**	***P* value**	R **pearson**	**Negative**	**Positive**	***P* value**	R **pearson**
Age (n = 25)								
**≥ 40 ≤ 60**<br>**> 60**	1 (12.5%)<br>5 (29.4%)	7 (87.5%)<br>12 (70.6%)	0.356	–0.185	1 (12.5%)<br>2 (11.8%)	7 (87.5%)<br>15 (88.2%)	0.958	0.011
Menopause (n = 25)								
**Pre-**<br>**Post-**	1 (20%)<br>5 (21.7%)	1 (50%)<br>18 (88.3%)	0.369	0.180	1 (50%)<br>2 (8.7%)	1 (50%)<br>21 (91.3%)	0.085	0.345
Histotype (n = 25)								
**IDC**<br>**ILC**<br>**MDLC**<br>**TC**	4 (33.3%)<br>0 (0%)<br>1 (33.3%)<br>1 (50%)	8 (76.7%)<br>8 (100%)<br>2 (76.7%)<br>1 (50%)	0.263	–0.059	1 (8.3%)<br>1 (12.5%)<br>0 (0%)<br>1 (50%)	11 (11.7%)<br>7 (87.5%)<br>3 (100%)<br>1 (50%)	0.348	–0.254
Size (n = 25)								
**≤ 2 cm**<br>**> 2 ≤ 5**<br>**> 5**	5 (23.8%)<br>1 (25%)<br>0 (0%)	16 (76.2%)<br>3 (75%)<br>0 (0%)	0.959	–0.010	3 (14.3%)<br>0 (0%)<br>0 (0%)	18 (85.7%)<br>4 (100%)<br>0 (0%)	0.420	0.161
LNM (n = 25)								
**Negative**<br>**Positive**	3 (21.4%)<br>3 (27.3%)	11 (78.6%)<br>8 (82.7%)	0.734	–0.068	2 (14.3%)<br>1 (9.1%)	12 (85.7%)<br>10 (90.9%)	0.692	0.079
Metastasis (n = 25)								
**Negative**<br>**Positive**	4 (26.7%)<br>2 (20%)	11 (77.3%)<br>8 (80%)	0.702	0.076	2 (13.3%)<br>1 (10%)	13 (86.7%)<br>9 (90%)	0.802	0.050
Grade (n = 25)								
**G1**<br>**G2**<br>**G3**	0 (0%)<br>1 (12.5%)<br>5 (29.4%)	0 (0%)<br>7 (87.5%)<br>12 (70.6%)	0.356	–0.185	0 (0%)<br>1 (12.5%)<br>2 (11.8%)	0 (0%)<br>7 (87.5%)<br>15 (88.2%)	0.958	0.011
Ki67 (n = 25)								
**≤ 30%**<br>**> 30%**	6 (27.3%)<br>0 (0%)	16 (82.7%)<br>3 (100%)	0.299	0.208	3 (41.2%)<br>0 (0%)	19 (58.8%)<br>3 (100%)	0.495	0.136
ErbB2 Status (n = 25)								
**0**<br>**1**<br>**2**<br>**3**	5 (29.4%)<br>0 (0%)<br>1 (100%)<br>0 (0%)	12 (70,6%)<br>5 (100%)<br>0 (0%)<br>1 (100%)	0.159	0.063	3 (17.6%)<br>0 (0%)<br>0 (0%)<br>0 (0%)	14 (82.4%)<br>5 (100%)<br>1 (100%)<br>1 (100%)	0.703	0.207
BMI (n = 22)								
**< 30**<br>**≥ 30**	3 (30%)<br>3 (25%)	7 (70%)<br>9 (75%)	0.793	0.056	1 (10%)<br>2 (16.7%)	9 (90%)<br>10 (83.3%)	0.650	–0.097
Diabetes (n = 25)								
**No**<br>**Yes**	4 (21%)<br>2 (33.3%)	15 (79%)<br>4 (66.7%)	0.539	–0.123	1 (5.3%)<br>2 (33.3%)	18 (94.7%)<br>4 (66.7%)	0.065	–0.369

Glycaemia (n = 25)								
**< 110**<br>**≥ 110 < 126**<br>**≥ 126**	3 (18.8%)<br>2 (50%)<br>1 (20%)	13 (81.2%)<br>2 (50%)<br>4 (80%)	0.413	–0.075	1 (6.3%)<br>1 (25%)<br>1 (20%)	15 (93.7%)<br>3 (75%)<br>4 (80%)	0.428	–0.202

Patients' mean age was 61 years (40–77). 2 patients (8%) were pre- menopausal and 23 (92%) post-menopausal. The patients had cancer of different histotype: 12 invasive ductal carcinoma (IDC), 8 invasive lobular carcinoma (ILC), 3 mixed ductal and lobular carcinoma (MDLC) and 2 tubular carcinoma (TC). Tumors larger than 2 cm occurred in 16% (4/25) of patients. 68% (17/25) of patients showed a tumor with poorly differentiated cells (grade 3), while only 32% (8/25) had tumor with moderately differentiated cells (grade 2). None of the tumors was of grade 1. Metastatic lymph nodes (LMN) were found in 44% (11/25) of patients at surgery and 40% (10/25) of patients developed distant metastases. The expression of the proliferation factor Ki67 was high (> 30%) in 12% (3/25), and low (≤ 30%) in 88% (22/25) of specimens. 17 of 25 (68%) patients showed a ErbB2 score of 0, 5 (20%) of 1+, 1 (4%) of 2+ and 1 (4%) of 3+. 24% (6/25) of patients were diabetic and 54,5% (12/22) had a BMI greater than 30; the BMI of 3 patients was not detectable.
